# Confocal laser endomicroscopy under propofol‐based sedation for early gastric cancer and pre‐cancerous lesions is associated with better diagnostic accuracy: a retrospective cohort study in China

**DOI:** 10.1186/s12871-021-01312-x

**Published:** 2021-03-30

**Authors:** Lihua Chu, Jialian Zhao, Cheng Sheng, Min Yue, Feifei Wang, Shengwen Song, Baoli Cheng, Guohao Xie, Xiangming Fang

**Affiliations:** 1 Department of Anesthesiology, the First Affiliated Hospital, College of Medicine, Zhejiang University, Zhejiang Hangzhou, China; 2 Department of Anesthesiology, the Children’s Hospital, College of Medicine, Zhejiang University, Hangzhou, China; 3 Department of Anesthesiology, the First Affiliated Hospital, College of Medicine, Zhejiang University, Beilun District, Ningbo, China; 4 Department of Gastroenterology, the First Affiliated Hospital, College of Medicine, Zhejiang University, Hangzhou, China; 5 Department of Intensive Care Unit, the First Affiliated Hospital, College of Medicine, Zhejiang University, Hangzhou, China

**Keywords:** Propofol‐based sedation, Confocal laser endomicroscopy, Early gastric cancer, Precancerous lesions

## Abstract

**Background:**

Confocal laser endomicroscopy (CLE) has advantages in detecting gastric neoplastic lesions, meanwhile it requires strict patient cooperation. Sedation could improve patient cooperation and quality of endoscopy. However, sedation is still not very popular in some resource-limited countries and regions. The purpose of this study was to compare propofol-based sedated versus un-sedated CLE in the value of diagnosing early gastric cancer (EGC) and precancerous lesions.

**Methods:**

A retrospective, cohort, single center study of 226 patients who underwent CLE between January 1, 2015 and December 31, 2017 was performed. Patients enrolled were allocated into the propofol-based sedated group (n = 126) and the un-sedated group (n = 100). The comparison of validity and reliability of CLE for identifying EGC and precancerous lesions between the two groups was performed through analyzing CLE diagnosis and pathological diagnosis. Reporting followed the STROBE guidelines.

**Results:**

The area under receiver operating characteristic curve (AUROC) of diagnosing EGC in the sedated group was 0.97 (95 % CI: 0.95 to 0.99), which was higher than that in the un-sedated group (0.88 (95 % CI: 0.80 to 0.97), *P* = 0.0407). CLE with sedation performed better than without sedation in diagnosing intraepithelial neoplasia and intestinal metaplasia (*P* = 0.0008 and *P* = 0.0001, respectively). For patients considered as high-grade intraepithelial neoplasia or EGC by endoscopists, they would not get biopsy during CLE but receive endoscopic submucosal dissection (ESD) subsequently, and the misdiagnosis rate of CLE was 0 % in the sedated group and 27.59 % (95 % CI: 10.30–44.91 %) in the un-sedated group (*P* = 0.006).

**Conclusions:**

Propofol based sedation was associated with improved diagnostic value of CLE for detecting EGC as well as precancerous lesions (intraepithelial neoplasia OR intestinal metaplasia).

## Background

Gastric cancer is one of the most common malignancy and leading causes of cancer mortality in the world [1–3], especially in East Asian countries [4, 5]. Detecting gastric malignancy at an early stage is vitally important, since early gastric cancer (EGC) may be curable, with reported 5-year survival rate of more than 90 % [6]. Advanced gastric cancer usually has poor prognosis [6].

As the most useful tools for screening gastric cancer, a number of modern endoscopy devices and techniques, like confocal laser endomicroscopy (CLE), magnifying endoscopy and narrow-band image, have been developed to fulfill different diagnostic demands. Among these, CLE has advantages in detecting EGC and pre-malignant lesions, since it can provide a direct histological observation of the cells and subcellular regions in vivo, as well as demonstrate mucosal changes that cannot be detected by white light endoscopy (WLE) [7].

Sedation in gastroscopy is widely accepted in high-income countries, which are found to be associated with increased patient comfort and reduced complications related with poor patient cooperation, though it causes additional medical resource consumption [8]. As CLE procedures usually require prolonged endoscopy time and better patient cooperation, sedation is considered even indispensable in these patients [9, 10]. However, whether sedation is associated with improved diagnostic quality of CLE is not well understood. Thus, evaluating the impact of sedation on CLE outcomes is still of value, especially in resource-limited countries and regions, such as China. Therefore, we conducted this retrospective, cohort study, to compare the diagnostic value for gastric superficial lesions, including EGC, intraepithelial neoplasia, and intestinal metaplasia between propofol based sedated and un-sedated CLE in a university hospital within China.

## Methods

### Study Design

This was a single-center, retrospective, cohort study, analyzing outpatient probe-based CLE database and histopathology reports from the Department of Endoscopy Unit, the First Affiliated hospital, College of Medicine, Zhejiang University between January 1, 2015 and December 31, 2017. The research protocol was reviewed and accepted by the research ethics committee of the First Affiliated Hospital, College of Medicine, Zhejiang University on June 13, 2018 (Reference Number: 750/2017). The study followed the reporting guideline-Strengthening the Reporting of Observational Studies in Epidemiology (STROBE).

### Participants

Patients enrolled were allocated into two groups according to their anesthesia type. Patients in the sedated group received a combination of propofol-based sedation and lidocaine-based pharyngeal anesthesia, while patients in the un-sedated group received lidocaine-based pharyngeal anesthesia only. Reports on juvenile patients (<18 years old), or patients with advanced gastric cancer were excluded.

### Anesthetic procedure

For all patients, after arrival, the electrocardiogram, non-invasive blood pressure and oxygen saturation were monitored. Lidocaine Hydrochloride mucilage (0.2 g/10ml; Jiangsu Jichuan Pharmaceutical Co., Jiangsu, China) was administered orally 5 min before the beginning of CLE for pharyngeal anesthesia. For patients with sedated CLE, a 20-gauge cannula was placed in a vein in the forearm, and sedation was induced with propofol (0.5 g/50ml; Xian Libang Pharmaceutical Co., Shanxi, China) 1.5-2 mg/kg intravenously. Sedation maintained was using 5–8 mg/kg/h propofol. Patients in the un-sedated group received pharyngeal anesthesia only.

### CLE procedure

CLE was conducted by two endoscopists who were with at least 5 years’ experience in performing diagnostic CLE. The procedure involved the use of a cellvizio confocal miniprobe (CM-4880, GastroFlex™ UHD, Mauna Kea, Paris, France), and a contrast agent (5ml of 10 % fluorescein sodium; Alcon Laboratories,Inc. Fort Worth, USA).

 CLE for all participants were performed according to the standard protocol. Each CLE procedure needed to be performed with no less than 20 min. After a mucosal lesion was visualized by WLE, a total of 5ml 10 % fluorescein sodium was administered intravenously. To obtain controlled CLE images, the probe was first gently contacted to normal mucosa around the lesion, ideally showing regular round or oval glands with homogeneous epithelial cells. The probe was subsequently moved to suspicious lesion to obtain CLE image, and following it, biopsies were obtained from the area. If the lesion was considered as high-grade intraepithelial neoplasia (HGIN) or EGC by the two endoscopists under CLE, the endoscopists would not get biopsy during the CLE procedure, but suggest patients for an endoscopic submucosal dissection (ESD) directly [6].

### CLE criteria and histopathological criteria

The CLE criteria were based on the 2011 Miami classification [11]. Four CLE diagnoses were given through evaluating architecture of glands, cells and micro-vessels as follows: [1] normal mucosa or benign inflammatory lesions; [2] atrophy and/or intestinal metaplasia (IM); [3] intraepithelial neoplasia (IN), including low-grade intraepithelial neoplasia (LGIN) and HGIN; [4] cancer.

The histopathological diagnostic criteria were based on the Updated Sydney System for classification and grading of gastritis [12] and the WHO classification of tumors [7]. EGC is defined as carcinoma confined to the mucosa or submucosa, regardless of lymph node metastatic status. Surgical or endoscopic ablation was recommended for neoplastic lesions. Endoscopic resection was selected for HGIN or mucosal carcinoma. Surgery was used for incomplete endoscopic resection [6].

### Data Collection

Two trained research assistants collected the following data: patients’ characteristics, CLE reports by endoscopists, pathological reports by pathologists. They also collected the information of patients who were strongly suspected for HGIN or EGC and further received ESD. These patients did not get biopsy during the CLE procedure, but had pathological results of ESD specimens. So the data of CLE reports and pathological diagnoses of ESD specimens were collected.

The complications appeared during CLE procedure were also recorded. Major complications were defined as need for intubation, intensive-care unit admission, resuscitation and death. Minor complications were defined as respiratory depression (Oxygen Saturation (SaO_2_) <90 % >10 s), hypotension (drop in systolic blood pressure of >25 %), hypertension (raise in systolic blood pressure of >25 %), bradycardia (drop in heart rate of >20 %), or tachycardia (>100 bpm).

These data were entered into a Microsoft Excel 2013 (Microsoft Corporation, Washington, United States). Another two trained research assistants randomly extracted 10 % of them to check the completeness, accuracy, and relevance of the information.

### Statistical analysis

Enrolled patients were allocated into two groups: the sedated group and the un-sedated group. The diagnostic accuracy of intestinal metaplasia, intraepithelial neoplasia, gastric neoplasia lesions (IN OR EGC) and EGC that examined by CLE was compared between the two groups. Among them, IM and IN were defined as precancerous lesions.

No specific power calculation was performed, and the sample size of this study was determined by the number of patients recruited across site. Statistical analysis was performed by using the software STATA/MP 15.0 (Stata Inc., TX, USA). Numerical data were presented as numbers (percentage). The Pearsonχ^2^ test was used to examine the significance of the association between two variables in a contingency table. Variables with a normal distribution were presented as mean ± standard deviation (SD), and compared using analysis of *t* test. A *P* value of 0.05 (two-sided) was considered statistically significant.

The primary outcome was the comparison of the area under receiver operating characteristic curve (AUROC) of EGC and precancerous lesions that was diagnosed by CLE between the two groups. The gold standard for diagnosing EGC and precancerous lesions is the histopathological diagnosis. The nonparametric analysis was used for two-group comparison in AUROC.

The second outcome was the misdiagnosis rate of CLE. For patients without biopsy during CLE and received an ESD subsequently, the misdiagnosis rate of CLE was analyzed according to their pathological results of ESD specimens.

Another second outcome was the comparison of sensitivity, specificity, positive predictive value (PPV), negative predictive value (NPV) and kappa (κ) value along with bionormal 95 % confidence intervals(95 % CI) between the two groups. Agreement was regarded as poor withκvalue below 0.4, good withκ value between 0.4 and 0.75, and excellent withκ value over 0.75.

## Results

Between January 1, 2015 and December 31, 2017, a total of 253 patients were eligible for study analysis, of which 27 (10 in the sedated group, 17 in the un-sedated group) was excluded because of the missing pathological data; the remaining 226 (126 in the sedated group, 100 in the un-sedated group) were analyzed. In this study, the most severe abnormality was used as each patient’s diagnosis. For example, intestinal metaplasia associated with intraepithelial neoplasia was considered as intraepithelial neoplasia. When referring to histopathology, in the sedated group, 16 (12.70 %) patients were diagnosed as EGC, 27 (21.43 %) patients were diagnosed as intraepithelial neoplasia, 55 (43.65 %) patients were diagnosed as intestinal metaplasia and 28 (22.22 %) patients were diagnosed as normal mucosa or benign inflammatory lesions. In the un-sedated group, 18 (18.00 %) patients were diagnosed as EGC, 17 (17.00 %) patients were diagnosed as intraepithelial neoplasia, 36 (36.00 %) patients were diagnosed as intestinal metaplasia and 29 (29.00 %) patients were diagnosed as normal mucosa or benign inflammatory lesions. Detailed characteristics of the two groups are presented in Table 1.

**Table 1:** Patients’ demographics

	The sedated group	The un-sedated group
Total number	126	100
Sex		
Male	77 (61.11 %)	59 (59.00 %)
Female	49 (38.89 %)	41 (41.00 %)
Age	58.10 ± 10.69	54.52 ± 11.61
Histopathological diagnosis		
Normal mucosa or benign inflammatory lesions	28 (22.22 %)	29 (29.00 %)
IM	55 (43.65 %)	36 (36.00 %)
IN	27 (21.43 %)	17 (17.00 %)
EGC	16 (12.70 %)	18 (18.00 %)

*IM* Intestinal metaplasia; *IN* Intraepithelial neoplasia; *EGC* Early gastric cancer

**Fig. 1:**
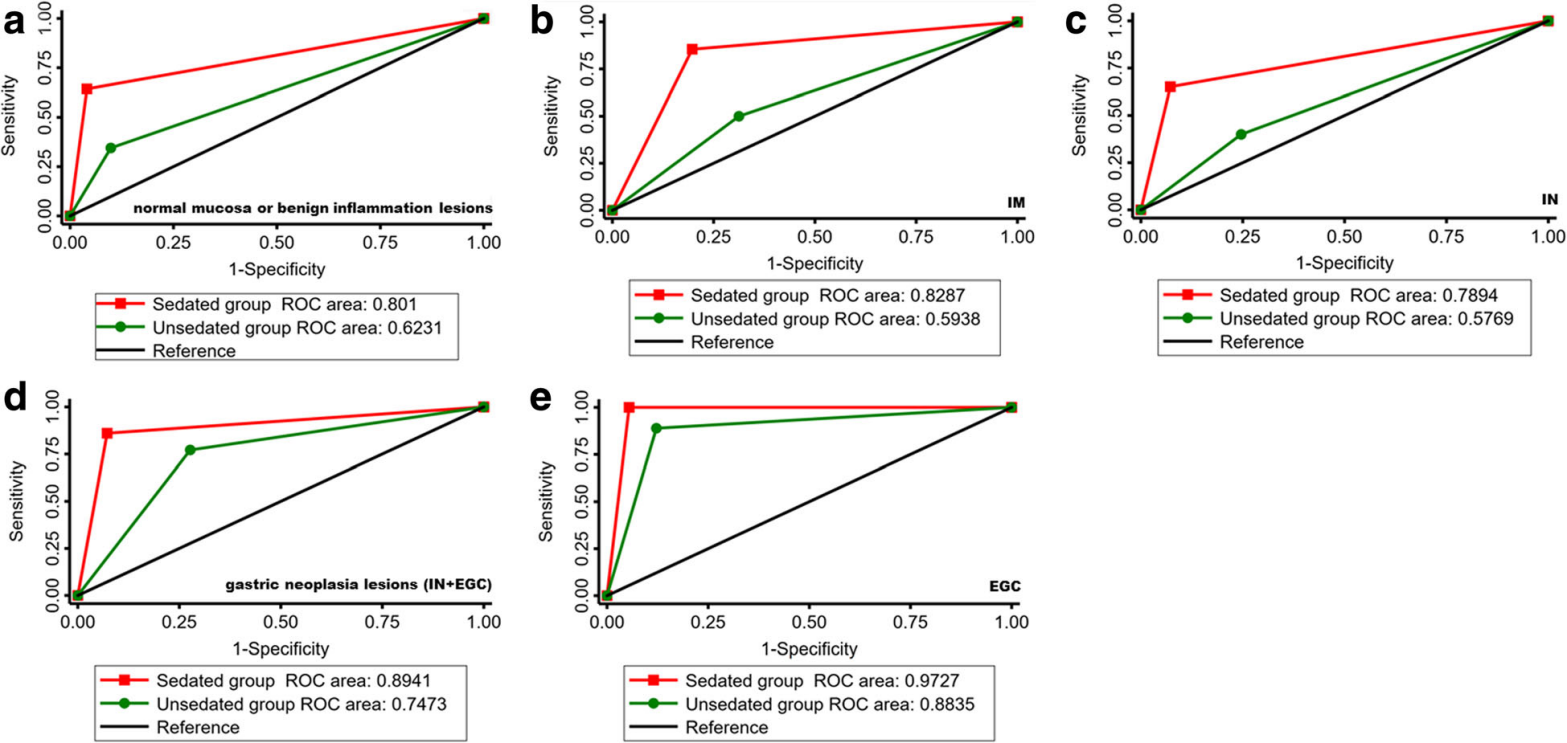
Comparison of AUROC of CLE for diagnosing different types of gastric lesions between the two groups. The value of CLE was better in the sedated CLE group than in the un-sedated CLE group in diagnosing normal mucosa or benign inflammation lesions (*P* = 0.0084) (**a**), intestinal metaplasia (*P* = 0.0001) (**b**), intraepithelial neoplasia (*P* = 0.0008) (**c**), gastric neoplasia lesions (IN + EGC) (*P* = 0.0073) (**d**) and EGC (*P* = 0.0407) (**e**). AUROC, The area under receiver operating characteristic curve; CLE, Confocal laser endomicroscopy; EGC, Early gastric cancer

The AUROC of CLE in diagnosing normal mucosa or benign inflammatory lesions in the sedated group was 0.80 (95 % CI: 0.71 to 0.89), which was higher than that in the un-sedated group (0.62 (95 % CI: 0.53 to 0.72), P = 0.0084) (Fig. 1a).

When referred to precancerous lesions, the sedated group had higher AUROC of CLE in diagnosing intestinal metaplasia than the un-sedated group (0.83 (95 % CI: 0.76 to 0.89) VS 0.59 (95 % CI: 0.49 to 0.69), *P* = 0.0001) (Fig. 1b). In diagnosing intraepithelial neoplasia, sedated CLE had higher AUROC (0.79 (95 % CI: 0.71 to 0.87)) than un-sedated CLE (0.58 (95 % CI: 0.48 to 0.67) (*P* = 0.0008) (Fig. 1c).

While testing the value of CLE in diagnosing neoplastic lesions (EGC + intraepithelial neoplasia), the AUROC in the sedated group was 0.89(0.83 to 0.95), which was higher than that in the un-sedated group (0.75(0.66 to 0.84)) (*P* = 0.0073) (Fig. 1d). The AUROC of CLE in diagnosing EGC in the sedated group was 0.97 (95 % CI: 0.95 to 0.99), and in the un-sedated group was 0.88 (95 % CI: 0.80 to 0.97) (*P* = 0.0407) (Fig. 1e).The sensitivity, specificity, PPV and NPV and κ of intestinal metaplasia, intraepithelial neoplasia, neoplastic lesions (EGC + intraepithelial neoplasia) and EGC were better in sedated CLE group than in un-sedated CLE group. Detailed information was listed in Table 2.

**Table 2:** Assessment of diagnostic value for gastric mucosal lesions based on CLE in the two groups

	Sensitivity (%,(CI))	Specificity (%,(CI))	PPV (%,(CI))	NPV (%,(CI))	κvalue
**Normal mucosa or benign inflammatory lesions**					
The sedated group	64.29(44.11 to 80.69)	95.92(89.28 to 98.68)	81.82(58.99 to 94.01)	90.38(82.62 to 95.04)	0.65
The un-sedated group	34.48(18.60 to 54.34) (18.60 to 54.33)	90.14(80.16 to 95.61)	58.82(33.45 to 80.57)	77.11(66.34 to 85.32)	0.28
**IM**					
The sedated group	85.55(72.78 to 93.07)	80.28(68.80 to 88.43)	77.05(64.20 to 86.46)	87.77(76.64 to 94.16)	0.65
The un-sedated group	50.00(33.22 to 66.78)	68.75(55.80 to 79.43)	47.37(31.31 to 63.95)	70.97(57.87 to 81.45)	0.19
**IN**					
The sedated group	65.11(49.01 to 78.55)	92.78(84.35 to 97.03)	82.35(64.83 to 92.61)	83.70(74.21 to 90.29)	0.61
The un-sedated group	40.00(24.35 to 57.79)	75.39(62.87 to 84.87)	46.67(28.80 to 65.36)	70.00(57.71 to 80.07)	0.16
**IN + EGC**					
The sedated group	86.05(71.37 to 94.20)	92.77(84.35 to 97.03)	86.05(71.37 to 94.20)	92.77(84.35 to 97.03)	0.79
The un-sedated group	77.14(59.45 to 88.96)	72.31(59.61 to 82.35)	60.00(44.37 to 73.94)	85.46(72.78 to 93.07)	0.46
**EGC**					
The sedated group	100(75.93 to 100)	94.55(88.02 to 97.76)	72.73(49.56 to 88.39)	100(95.56 to 100)	0.82
The un-sedated group	88.89(63.93 to 68.05)	87.80(78.27 to 93.68)	61.54(40.73 to 79.09)	97.30(89.69 to 99.53)	0.65

*IM* Intestinal metaplasia; *IN* Intraepithelial neoplasia; *EGC* Early gastric cancer

To those who received ESD, the agreement between main CLE finding and the ESD pathological diagnose was assessed. A total of 24 patients in the sedated group and 29 in the un-sedated group were suggested for ESD directly. The misdiagnosis rate of CLE in the sedated group was 0 %, and in the un-sedated group was 27.59 % (95 % CI: 10.30–44.91 %), which had significant difference (*P* = 0.006).

Minor complication of hypotension was found in two patients with sedated CLE. Three patients and one patient in the un-sedated group suffered tachycardia and hypertension, respectively. There were no major complications found in the two groups.

## Discussion

Our results showed that CLE with propofol-based sedation had remarkably better discrimination for diagnosing EGC and pre-malignant lesions (intestinal metaplasia and intraepithelial neoplasia) than that of un-sedated CLE. And for patients without biopsy during CLE procedure and received an ESD directly, the misdiagnosis rate of CLE was significantly lower in the sedated group than in the un-sedated group according to the final pathological results. To the best of our knowledge, this is the first study to demonstrate such a positive association between sedation and CLE outcomes.

Sedation is a drug-induced depression in the level of consciousness, which is recommended for GI endoscopy [13]. It could relieve the patients’ anxiety and discomfort, diminish the patients’ memory of the event and improve the outcome of examination [14]. Our study showed sedated CLE had a better diagnostic value for EGC than un-sedated CLE. The results of the sedated group were similar with the previous study, whose sensitivity and specificity of diagnosing EGC was 88.1 and 98.6 % respectively [6], but results in the un-sedated group seems not so satisfactory. This may due to three reasons: Firstly, patients with sedation are able to well tolerate the CLE procedure [13] and endure inflation of the stomach to a greater extent, compared to patients without sedation [15]; Secondly, adequate level of sedation in CLE may improve the efficiency and quality of the procedure by providing the endoscopist with optimal conditions for a thorough visualization, while eliminating any distraction due to an uncomfortable patient [16]; Thirdly, with sedation, the examining time can be prolonged without patient complaints if needed [15]. The operator of CLE would be more focused and confident during the examinations and in no hurry to finish the procedure.

Gastric cancer is believed to arise from a series of pre-malignant lesions, through a number of stages from chronic atrophic gastritis, by way of intestinal metaplasia, through LGIN and HGIN, up to cancer [17]. A large sample study showed that approximately 1 in 39 with intestinal metaplasia and 1 in 19 with dysplasia would progress to gastric cancer within 20 years [18]. Considering the higher incidence of intestinal metaplasia and intraepithelial neoplasia compared with EGC, especially in high-risk regions like China, it is desirable to explore whether CLE with sedation could improve the diagnostic value of identifying precancerous lesions [19]. The ROC curve analysis revealed that sedation could increase the AUROC of CLE diagnosing intestinal metaplasia and intraepithelial neoplasia. It is important because immediate diagnosis as well as precise biopsy can help endoscopist to make a quick decision for treatment, especially for intraepithelial neoplasia at a high grade.

In the current study, propofol based sedation was adopted, which makes endoscopy almost painless, with a very predictable, rapid recovery process and improved patient satisfaction [20, 21]. Compared with benzodiazepines and opioids, sedation with propofol can improve the quality of endoscopy, such as increasing the detecting rate of advanced lesions [22] and polyp [16]. Sedation is considered with an added risk of complications, but the complication and mortality rates of a prospective research, involving 191,242 endoscopies with propofol sedation, were 0.04 and 0.003 %, respectively [24]. In addition, a recent large multicenter registry study, with 300 000 patients enrolled, confirmed that severe acute sedation-related complications are rare during GI endoscopy with a very low mortality [25].

Although sedated endoscopy is widely accepted in high-income countries and regions [14], it is not so popular in resource-limited countries and regions [26]. For example, in the studied tertiary hospital, which is located in a wealthier area of China, the ratio of sedation in gastroscopy was only 43.2 % in 2018. The limited staffing of anesthesiologists is the main restriction factor. Although extensive data have demonstrated the safety and efficacy of non-anesthesiologist-administered propofol sedation, the American Society of Anesthesiologists continues to maintain that propofol sedation should be performed only by anesthesia providers [23, 27]. The economic factor also hinders the development of sedated CLE in China. Our study showed sedation could improve the diagnostic ability of EGC and pre-malignant lesions. Thus, it might be better for government to put more medical resources and provide more medical insurance supports in the field of sedated CLE. And the publicity of sedation should be further strengthened by the hospital administration as well as relevant medical departments.

Our study was limited by a few factors. Firstly, its retrospective study design was the main weakness. A prospective validation study is needed to confirm the association between sedation and improved quality of CLE. Furthermore, in the validation phase, we can also assess the safety and patients satisfactory of sedation. Secondly, the procedure time was not recorded in our databases. The procedure of CLE with propofol-based sedation might be prolonged for the patient’s great cooperation. The longer time might be correlated with the quality of mucosal inspection during CLE.

## Conclusions

In summary, this is the first study to validate the propofol-based sedation in improving the value of CLE in diagnosing EGC and precancerous lesions. Our results showed the improvement of validity and reliability of CLE in diagnosing gastric superficial cancerous and precancerous lesions though sedation. It indicates that, especially in resource-limited countries and regions, more patient undergoing CLE would benefit from sedation if more medical resources were put into this area.

## Data Availability

The datasets generated and analyzed during the present study are available from the corresponding author on reasonable request.

## Abbreviations

95% CI: 95 % confidence intervals
AUROC: Area under receiver operating characteristic curve
CLE: Confocal laser endomicroscopy
EGC: Early gastric cancer
ESD: Endoscopic submucosal dissection
HGIN: High-grade intraepithelial neoplasia
IM: Intestinal metaplasia
IN: Intraepithelial neoplasia
LGIN: Low-grade intraepithelial neoplasia
NPV: Negative predictive value
PPV: Positive predictive value
ROC: Receiver operating characteristic
SD: Standard deviation
WLE: White light endoscopy
